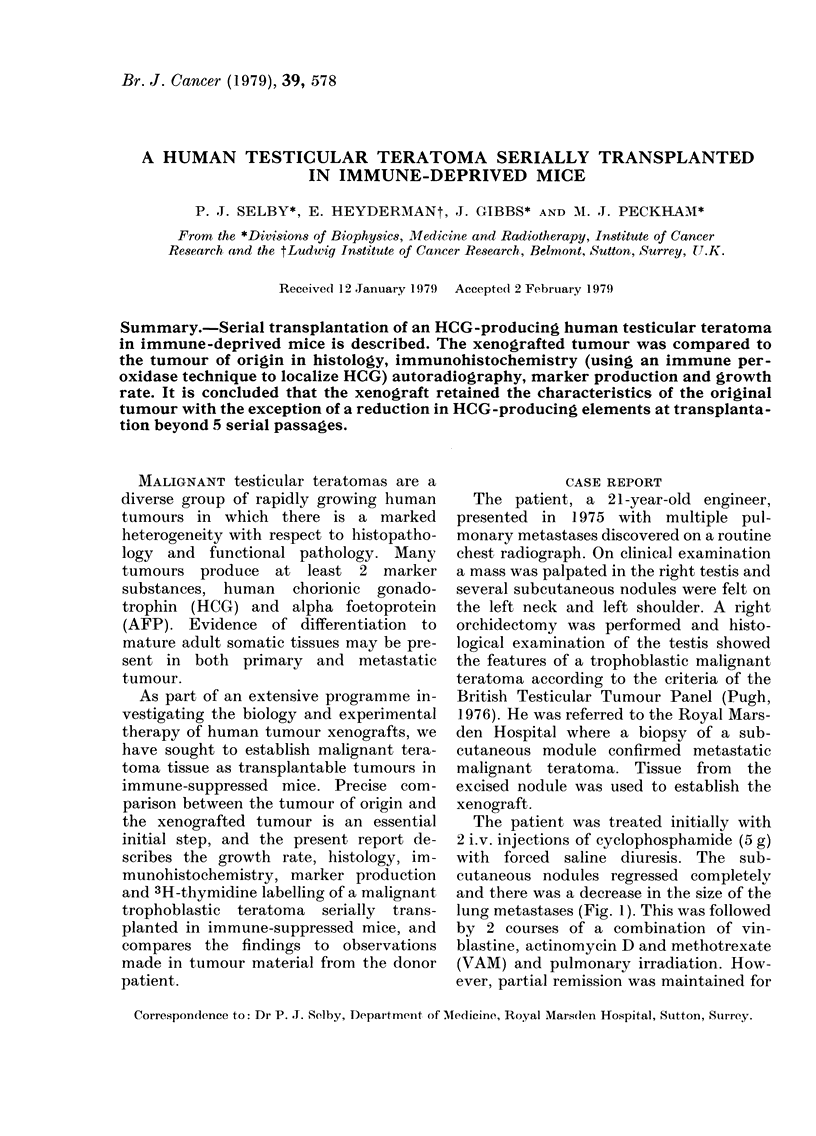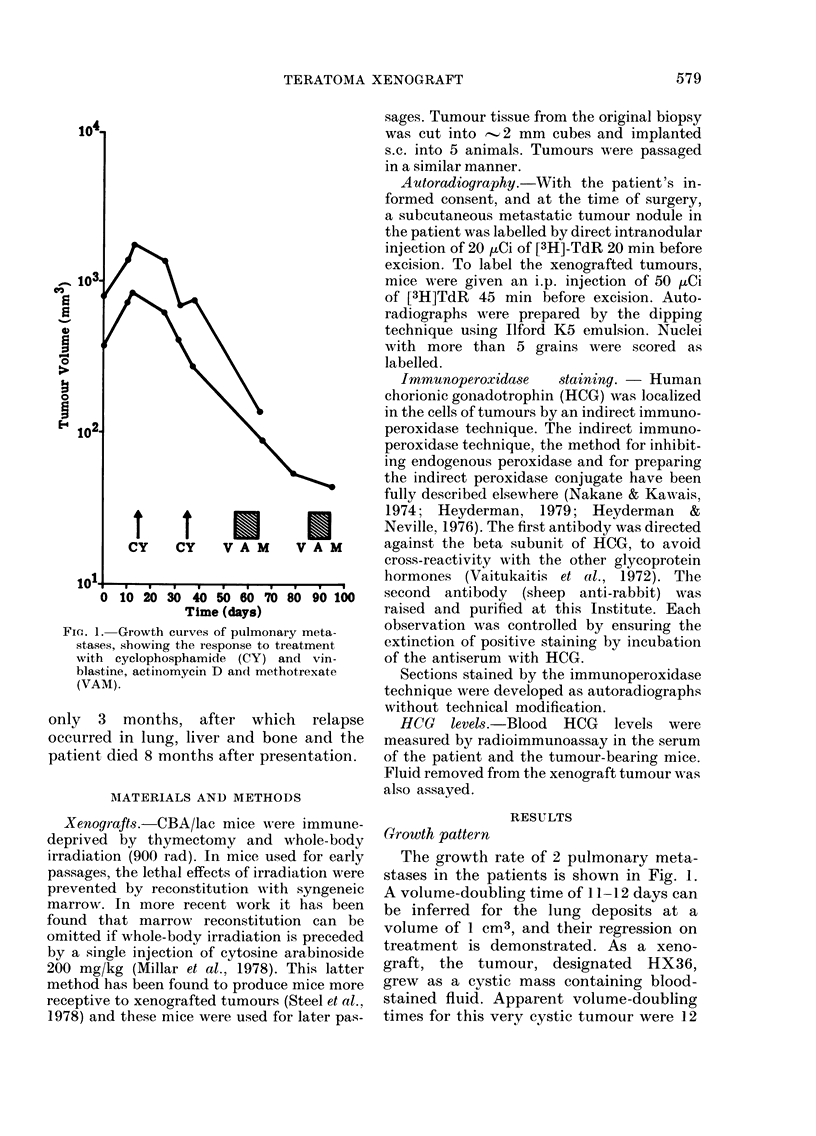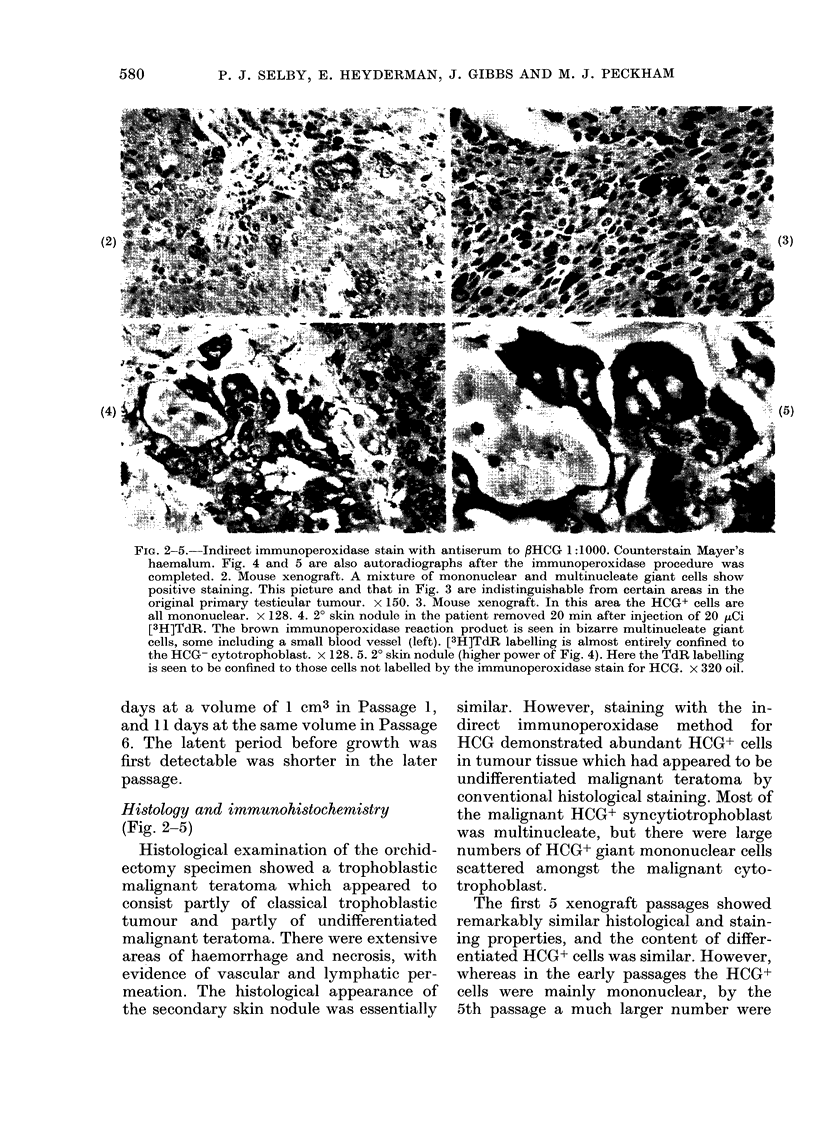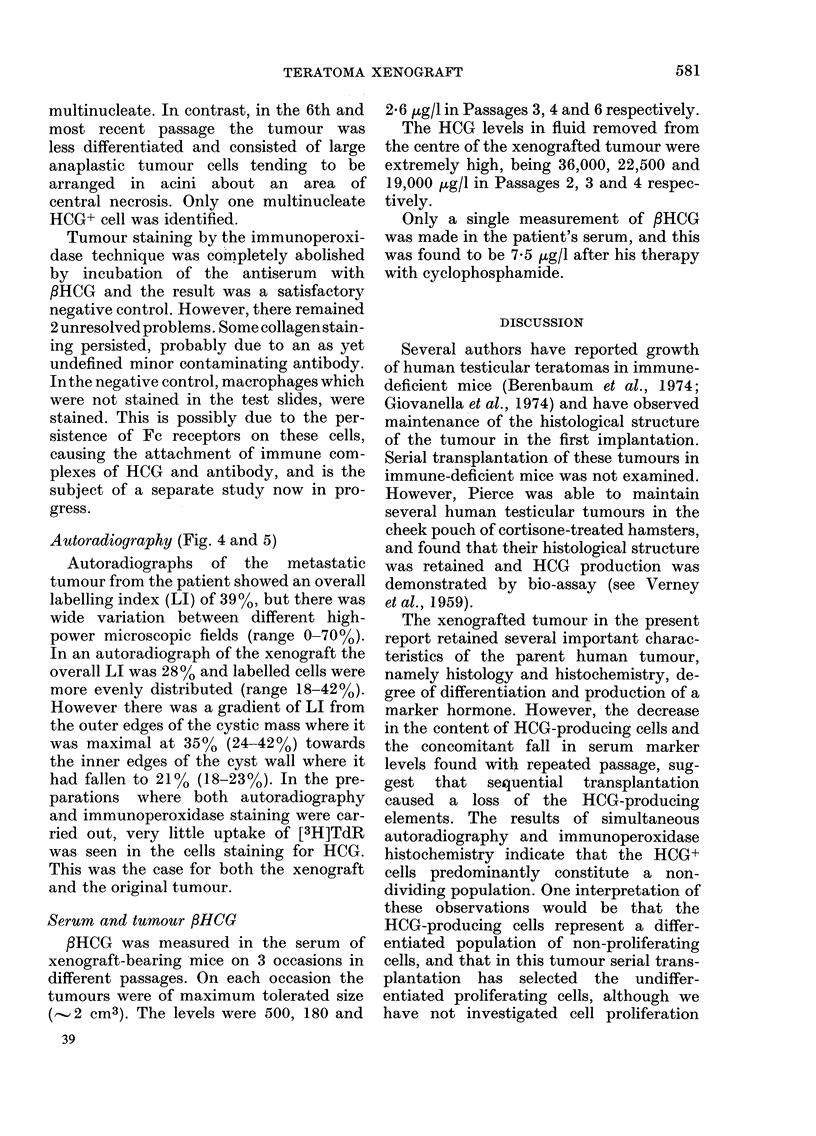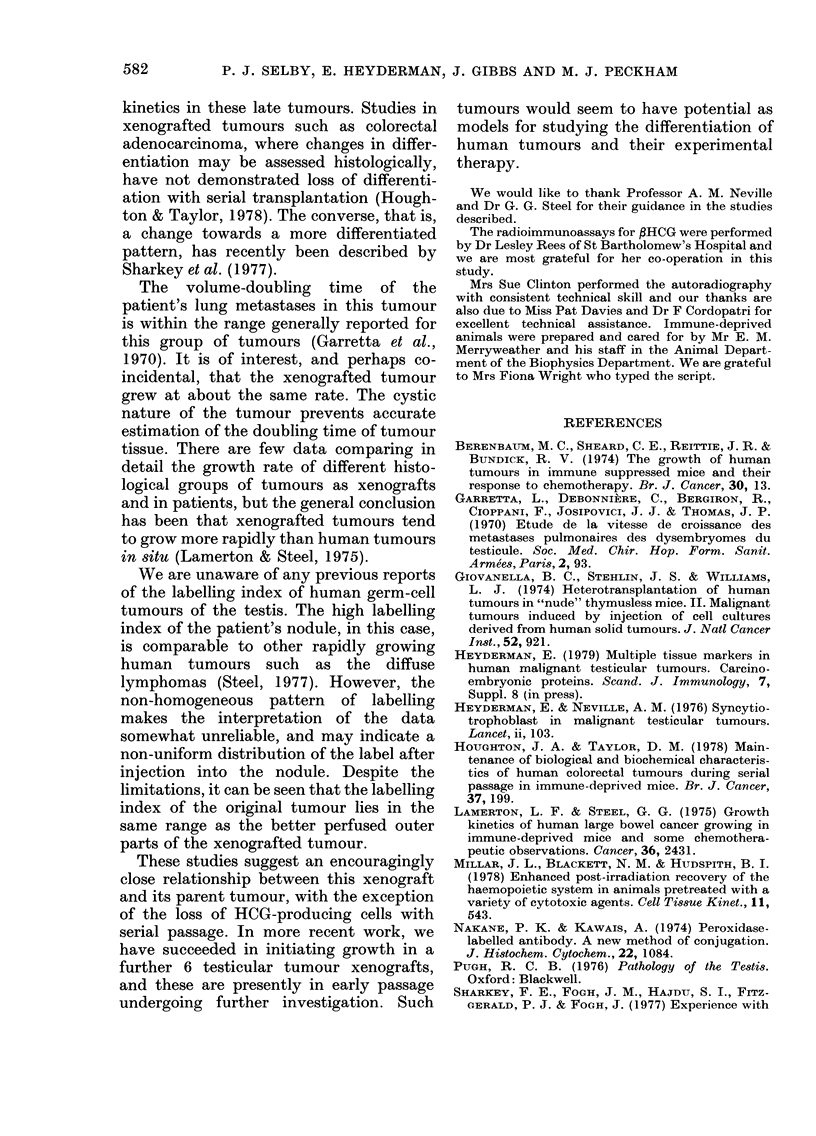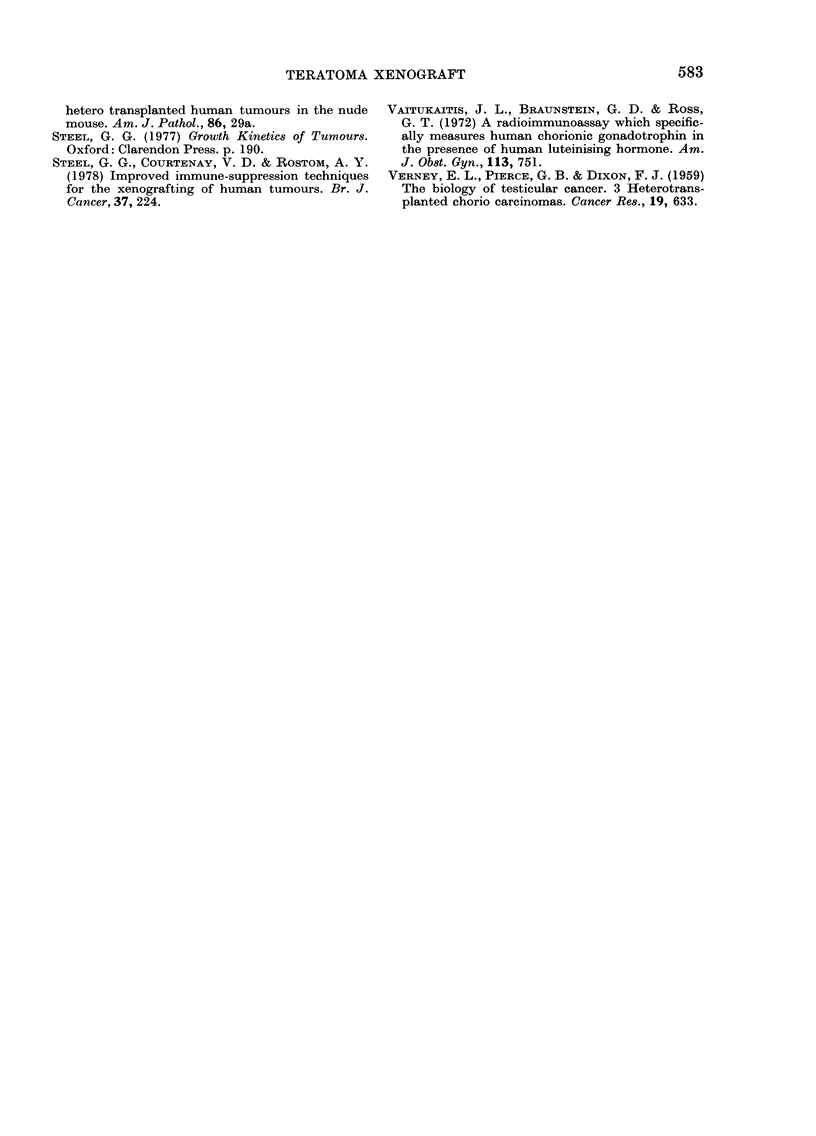# A human testicular teratoma serially transplanted in immune-deprived mice.

**DOI:** 10.1038/bjc.1979.104

**Published:** 1979-05

**Authors:** P. J. Selby, E. Heyderman, J. Gibbs, M. J. Peckham

## Abstract

**Images:**


*Br. J. Cancer* (1979), **39**, 578

### A HUMAN TESTICULAR TERATOMA SERIALLY TRANSPLANTED IN IMMUNE-DEPRIVED MICE

P. J. SELBY*, E. HEYDERMAN†, J. GIBBS* AND M. J. PECKHAM*

*From the *Divisions of Biophysics, Medicine and Radiotherapy, Institute of Cancer Research and the †Ludwig Institute of Cancer Research, Belmont, Sutton, Surrey, U.K.*

Received 12 January 1979 Accepted 2 February 1979

**Summary.—Serial transplantation of an HCG-producing human testicular teratoma in immune-deprived mice is described. The xenografted tumour was compared to the tumour of origin in histology, immunohistochemistry (using an immune peroxidase technique to localize HCG) autoradiography, marker production and growth rate. It is concluded that the xenograft retained the characteristics of the original tumour with the exception of a reduction in HCG-producing elements at transplantation beyond 5 serial passages.**

MALIGNANT testicular teratomas are a diverse group of rapidly growing human tumours in which there is a marked heterogeneity with respect to histopathology and functional pathology. Many tumours produce at least 2 marker substances, human chorionic gonadotrophin (HCG) and alpha foetoprotein (AFP). Evidence of differentiation to mature adult somatic tissues may be present in both primary and metastatic tumour.

As part of an extensive programme investigating the biology and experimental therapy of human tumour xenografts, we have sought to establish malignant teratoma tissue as transplantable tumours in immune-suppressed mice. Precise comparison between the tumour of origin and the xenografted tumour is an essential initial step, and the present report describes the growth rate, histology, immunohistochemistry, marker production and $^3$H-thymidine labelling of a malignant trophoblastic teratoma serially transplanted in immune-suppressed mice, and compares the findings to observations made in tumour material from the donor patient.

### CASE REPORT

The patient, a 21-year-old engineer, presented in 1975 with multiple pulmonary metastases discovered on a routine chest radiograph. On clinical examination a mass was palpated in the right testis and several subcutaneous nodules were felt on the left neck and left shoulder. A right orchidectomy was performed and histological examination of the testis showed the features of a trophoblastic malignant teratoma according to the criteria of the British Testicular Tumour Panel (Pugh, 1976). He was referred to the Royal Marsden Hospital where a biopsy of a subcutaneous module confirmed metastatic malignant teratoma. Tissue from the excised nodule was used to establish the xenograft.

The patient was treated initially with 2 i.v. injections of cyclophosphamide (5 g) with forced saline diuresis. The subcutaneous nodules regressed completely and there was a decrease in the size of the lung metastases (Fig. 1). This was followed by 2 courses of a combination of vinblastine, actinomycin D and methotrexate (VAM) and pulmonary irradiation. However, partial remission was maintained for

Correspondence to: Dr P. J. Selby, Department of Medicine, Royal Marsden Hospital, Sutton, Surrey.

FIG. 1.—Growth curves of pulmonary metastases, showing the response to treatment with cyclophosphamide (CY) and vinblastine, actinomycin D and methotrexate (VAM).

only 3 months, after which relapse occurred in lung, liver and bone and the patient died 8 months after presentation.

### MATERIALS AND METHODS

*Xenografts*.—CBA/lac mice were immune-deprived by thymectomy and whole-body irradiation (900 rad). In mice used for early passages, the lethal effects of irradiation were prevented by reconstitution with syngeneic marrow. In more recent work it has been found that marrow reconstitution can be omitted if whole-body irradiation is preceded by a single injection of cytosine arabinoside 200 mg/kg (Millar *et al.*, 1978). This latter method has been found to produce mice more receptive to xenografted tumours (Steel *et al.*, 1978) and these mice were used for later passages. Tumour tissue from the original biopsy was cut into ~2 mm cubes and implanted s.c. into 5 animals. Tumours were passaged in a similar manner.

*Autoradiography*.—With the patient's informed consent, and at the time of surgery, a subcutaneous metastatic tumour nodule in the patient was labelled by direct intranodular injection of 20 $\mu$Ci of [$^3$H]-TdR 20 min before excision. To label the xenografted tumours, mice were given an i.p. injection of 50 $\mu$Ci of [$^3$H]TdR 45 min before excision. Autoradiographs were prepared by the dipping technique using Ilford K5 emulsion. Nuclei with more than 5 grains were scored as labelled.

*Immunoperoxidase staining*. — Human chorionic gonadotrophin (HCG) was localized in the cells of tumours by an indirect immunoperoxidase technique. The indirect immunoperoxidase technique, the method for inhibiting endogenous peroxidase and for preparing the indirect peroxidase conjugate have been fully described elsewhere (Nakane & Kawais, 1974; Heyderman, 1979; Heyderman & Neville, 1976). The first antibody was directed against the beta subunit of HCG, to avoid cross-reactivity with the other glycoprotein hormones (Vaitukaitis *et al.*, 1972). The second antibody (sheep anti-rabbit) was raised and purified at this Institute. Each observation was controlled by ensuring the extinction of positive staining by incubation of the antiserum with HCG.

Sections stained by the immunoperoxidase technique were developed as autoradiographs without technical modification.

*HCG levels*.—Blood HCG levels were measured by radioimmunoassay in the serum of the patient and the tumour-bearing mice. Fluid removed from the xenograft tumour was also assayed.

### RESULTS

### *Growth pattern*

The growth rate of 2 pulmonary metastases in the patients is shown in Fig. 1. A volume-doubling time of 11–12 days can be inferred for the lung deposits at a volume of 1 cm$^3$, and their regression on treatment is demonstrated. As a xenograft, the tumour, designated HX36, grew as a cystic mass containing blood-stained fluid. Apparent volume-doubling times for this very cystic tumour were 12

(2) (3) (4) (5)

FIG. 2–5.—Indirect immunoperoxidase stain with antiserum to βHCG 1:1000. Counterstain Mayer's haemalum. Fig. 4 and 5 are also autoradiographs after the immunoperoxidase procedure was completed. 2. Mouse xenograft. A mixture of mononuclear and multinucleate giant cells show positive staining. This picture and that in Fig. 3 are indistinguishable from certain areas in the original primary testicular tumour. ×150. 3. Mouse xenograft. In this area the $HCG^+$ cells are all mononuclear. ×128. 4. 2° skin nodule in the patient removed 20 min after injection of 20 μCi [$^3$H]TdR. The brown immunoperoxidase reaction product is seen in bizarre multinucleate giant cells, some including a small blood vessel (left). [$^3$H]TdR labelling is almost entirely confined to the $HCG^-$ cytotrophoblast. ×128. 5. 2° skin nodule (higher power of Fig. 4). Here the TdR labelling is seen to be confined to those cells not labelled by the immunoperoxidase stain for HCG. ×320 oil.

days at a volume of 1 cm$^3$ in Passage 1, and 11 days at the same volume in Passage 6. The latent period before growth was first detectable was shorter in the later passage.

*Histology and immunohistochemistry* (Fig. 2–5)

Histological examination of the orchidectomy specimen showed a trophoblastic malignant teratoma which appeared to consist partly of classical trophoblastic tumour and partly of undifferentiated malignant teratoma. There were extensive areas of haemorrhage and necrosis, with evidence of vascular and lymphatic permeation. The histological appearance of the secondary skin nodule was essentially similar. However, staining with the indirect immunoperoxidase method for HCG demonstrated abundant $HCG^+$ cells in tumour tissue which had appeared to be undifferentiated malignant teratoma by conventional histological staining. Most of the malignant $HCG^+$ syncytiotrophoblast was multinucleate, but there were large numbers of $HCG^+$ giant mononuclear cells scattered amongst the malignant cytotrophoblast.

The first 5 xenograft passages showed remarkably similar histological and staining properties, and the content of differentiated $HCG^+$ cells was similar. However, whereas in the early passages the $HCG^+$ cells were mainly mononuclear, by the 5th passage a much larger number were multinucleate. In contrast, in the 6th and most recent passage the tumour was less differentiated and consisted of large anaplastic tumour cells tending to be arranged in acini about an area of central necrosis. Only one multinucleate $HCG^+$ cell was identified.

Tumour staining by the immunoperoxidase technique was completely abolished by incubation of the antiserum with βHCG and the result was a satisfactory negative control. However, there remained 2 unresolved problems. Some collagen staining persisted, probably due to an as yet undefined minor contaminating antibody. In the negative control, macrophages which were not stained in the test slides, were stained. This is possibly due to the persistence of Fc receptors on these cells, causing the attachment of immune complexes of HCG and antibody, and is the subject of a separate study now in progress.

*Autoradiography* (Fig. 4 and 5)

Autoradiographs of the metastatic tumour from the patient showed an overall labelling index (LI) of 39%, but there was wide variation between different high-power microscopic fields (range 0–70%). In an autoradiograph of the xenograft the overall LI was 28% and labelled cells were more evenly distributed (range 18–42%). However there was a gradient of LI from the outer edges of the cystic mass where it was maximal at 35% (24–42%) towards the inner edges of the cyst wall where it had fallen to 21% (18–23%). In the preparations where both autoradiography and immunoperoxidase staining were carried out, very little uptake of $[^3H]TdR$ was seen in the cells staining for HCG. This was the case for both the xenograft and the original tumour.

*Serum and tumour βHCG*

βHCG was measured in the serum of xenograft-bearing mice on 3 occasions in different passages. On each occasion the tumours were of maximum tolerated size ($\sim 2$ cm$^3$). The levels were 500, 180 and 2·6 μg/l in Passages 3, 4 and 6 respectively.

The HCG levels in fluid removed from the centre of the xenografted tumour were extremely high, being 36,000, 22,500 and 19,000 μg/l in Passages 2, 3 and 4 respectively.

Only a single measurement of βHCG was made in the patient's serum, and this was found to be 7·5 μg/l after his therapy with cyclophosphamide.

### DISCUSSION

Several authors have reported growth of human testicular teratomas in immune-deficient mice (Berenbaum *et al.*, 1974; Giovanella *et al.*, 1974) and have observed maintenance of the histological structure of the tumour in the first implantation. Serial transplantation of these tumours in immune-deficient mice was not examined. However, Pierce was able to maintain several human testicular tumours in the cheek pouch of cortisone-treated hamsters, and found that their histological structure was retained and HCG production was demonstrated by bio-assay (see Verney *et al.*, 1959).

The xenografted tumour in the present report retained several important characteristics of the parent human tumour, namely histology and histochemistry, degree of differentiation and production of a marker hormone. However, the decrease in the content of HCG-producing cells and the concomitant fall in serum marker levels found with repeated passage, suggest that sequential transplantation caused a loss of the HCG-producing elements. The results of simultaneous autoradiography and immunoperoxidase histochemistry indicate that the $HCG^+$ cells predominantly constitute a non-dividing population. One interpretation of these observations would be that the HCG-producing cells represent a differentiated population of non-proliferating cells, and that in this tumour serial transplantation has selected the undifferentiated proliferating cells, although we have not investigated cell proliferation kinetics in these late tumours. Studies in xenografted tumours such as colorectal adenocarcinoma, where changes in differentiation may be assessed histologically, have not demonstrated loss of differentiation with serial transplantation (Houghton & Taylor, 1978). The converse, that is, a change towards a more differentiated pattern, has recently been described by Sharkey *et al.* (1977).

The volume-doubling time of the patient's lung metastases in this tumour is within the range generally reported for this group of tumours (Garretta *et al.*, 1970). It is of interest, and perhaps coincidental, that the xenografted tumour grew at about the same rate. The cystic nature of the tumour prevents accurate estimation of the doubling time of tumour tissue. There are few data comparing in detail the growth rate of different histological groups of tumours as xenografts and in patients, but the general conclusion has been that xenografted tumours tend to grow more rapidly than human tumours *in situ* (Lamerton & Steel, 1975).

We are unaware of any previous reports of the labelling index of human germ-cell tumours of the testis. The high labelling index of the patient's nodule, in this case, is comparable to other rapidly growing human tumours such as the diffuse lymphomas (Steel, 1977). However, the non-homogeneous pattern of labelling makes the interpretation of the data somewhat unreliable, and may indicate a non-uniform distribution of the label after injection into the nodule. Despite the limitations, it can be seen that the labelling index of the original tumour lies in the same range as the better perfused outer parts of the xenografted tumour.

These studies suggest an encouragingly close relationship between this xenograft and its parent tumour, with the exception of the loss of HCG-producing cells with serial passage. In more recent work, we have succeeded in initiating growth in a further 6 testicular tumour xenografts, and these are presently in early passage undergoing further investigation. Such tumours would seem to have potential as models for studying the differentiation of human tumours and their experimental therapy.

We would like to thank Professor A. M. Neville and Dr G. G. Steel for their guidance in the studies described.

The radioimmunoassays for βHCG were performed by Dr Lesley Rees of St Bartholomew's Hospital and we are most grateful for her co-operation in this study.

Mrs Sue Clinton performed the autoradiography with consistent technical skill and our thanks are also due to Miss Pat Davies and Dr F Cordopatri for excellent technical assistance. Immune-deprived animals were prepared and cared for by Mr E. M. Merryweather and his staff in the Animal Department of the Biophysics Department. We are grateful to Mrs Fiona Wright who typed the script.

### REFERENCES

Berenbaum, M. C., Sheard, C. E., Reittie, J. R. & Bundick, R. V. (1974) The growth of human tumours in immune suppressed mice and their response to chemotherapy. *Br. J. Cancer*, **30**, 13.

Garretta, L., Debonnière, C., Bergiron, R., Cioppani, F., Josipovici, J. J. & Thomas, J. P. (1970) Etude de la vitesse de croissance des metastases pulmonaires des dysembryomes du testicule. *Soc. Med. Chir. Hop. Form. Sanit. Armées, Paris*, **2**, 93.

Giovanella, B. C., Stehlin, J. S. & Williams, L. J. (1974) Heterotransplantation of human tumours in "nude" thymusless mice. II. Malignant tumours induced by injection of cell cultures derived from human solid tumours. *J. Natl Cancer Inst.*, **52**, 921.

Heyderman, E. (1979) Multiple tissue markers in human malignant testicular tumours. Carcinoembryonic proteins. *Scand. J. Immunology*, **7**, Suppl. 8 (in press).

Heyderman, E. & Neville, A. M. (1976) Syncytiotrophoblast in malignant testicular tumours. *Lancet*, ii, 103.

Houghton, J. A. & Taylor, D. M. (1978) Maintenance of biological and biochemical characteristics of human colorectal tumours during serial passage in immune-deprived mice. *Br. J. Cancer*, **37**, 199.

Lamerton, L. F. & Steel, G. G. (1975) Growth kinetics of human large bowel cancer growing in immune-deprived mice and some chemotherapeutic observations. *Cancer*, **36**, 2431.

Millar, J. L., Blackett, N. M. & Hudspith, B. I. (1978) Enhanced post-irradiation recovery of the haemopoietic system in animals pretreated with a variety of cytotoxic agents. *Cell Tissue Kinet.*, **11**, 543.

Nakane, P. K. & Kawais, A. (1974) Peroxidase-labelled antibody. A new method of conjugation. *J. Histochem. Cytochem.*, **22**, 1084.

Pugh, R. C. B. (1976) *Pathology of the Testis.* Oxford: Blackwell.

Sharkey, F. E., Fogh, J. M., Hajdu, S. I., Fitzgerald, P. J. & Fogh, J. (1977) Experience with hetero transplanted human tumours in the nude mouse. *Am. J. Pathol.*, **86**, 29a.

STEEL, G. G. (1977) *Growth Kinetics of Tumours.* Oxford: Clarendon Press. p. 190.

STEEL, G. G., COURTENAY, V. D. & ROSTOM, A. Y. (1978) Improved immune-suppression techniques for the xenografting of human tumours. *Br. J. Cancer*, **37**, 224.

VAITUKAITIS, J. L., BRAUNSTEIN, G. D. & ROSS, G. T. (1972) A radioimmunoassay which specifically measures human chorionic gonadotrophin in the presence of human luteinising hormone. *Am. J. Obst. Gyn.*, **113**, 751.

VERNEY, E. L., PIERCE, G. B. & DIXON, F. J. (1959) The biology of testicular cancer. 3 Heterotransplanted chorio carcinomas. *Cancer Res.*, **19**, 633.